# Multiplex Serology for Sensitive and Specific Flavivirus IgG Detection: Addition of Envelope Protein Domain III to NS1 Increases Sensitivity for Tick-Borne Encephalitis Virus IgG Detection

**DOI:** 10.3390/v16020286

**Published:** 2024-02-13

**Authors:** Coralie Valle, Sandhya Shrestha, Gert-Jan Godeke, Marieke N. Hoogerwerf, Johan Reimerink, Dirk Eggink, Chantal Reusken

**Affiliations:** 1 Centre for Infectious Disease Control, National Institute for Public Health and the Environment, 3721 MA Bilthoven, The Netherlandsmarieke.hoogerwerf@rivm.nl (M.N.H.); johan.reimerink@rivm.nl (J.R.);; 2 Unité des Virus Emergents (UVE), Aix-Marseille Université, IRD 190, Inserm 1207, 13005 Marseille, France

**Keywords:** multiplex protein array, tick-borne encephalitis, flavivirus, NS1, EDIII, serology

## Abstract

Tick-borne encephalitis is a vaccine-preventable disease of concern for public health in large parts of Europe, with EU notification rates increasing since 2018. It is caused by the orthoflavivirus tick-borne encephalitis virus (TBEV) and a diagnosis of infection is mainly based on serology due to its short viremic phase, often before symptom onset. The interpretation of TBEV serology is hampered by a history of orthoflavivirus vaccination and by previous infections with related orthoflaviviruses. Here, we sought to improve TBEV sero-diagnostics using an antigen combination of in-house expressed NS1 and EDIII in a multiplex, low-specimen-volume set-up for the detection of immune responses to TBEV and other clinically important orthoflaviviruses (i.e., West Nile virus, dengue virus, Japanese encephalitis virus, Usutu virus and Zika virus). We show that the combined use of NS1 and EDIII results in both a specific and sensitive test for the detection of TBEV IgG for patient diagnostics, vaccination responses and in seroprevalence studies. This novel approach potentially allows for a low volume-based, simultaneous analysis of IgG responses to a range of orthoflaviviruses with overlapping geographic circulations and clinical manifestations.

## 1. Introduction

Over the past decades, the geographic distribution of viruses transmitted by arthropods like mosquitos and ticks, grouped under the term “Arboviruses”, has expanded, and the burden of associated disease is increasing worldwide. Tick-borne encephalitis virus (TBEV) is an important arbovirus for public health, affecting the human central nervous system (CNS), and it is now widespread in Europe and Asia [1]. Between 2000 and 4000 TBEV infections are reported every year in Europe, and TBEV is of concern for a significant part of Europe [2]. Autochthonous cases of tick-borne encephalitis (TBE, the disease caused by TBEV), were identified for the first time in the Netherlands in 2016. Since then, 16 autochthonous cases have been identified [3,4]. 

TBEV is transmitted to humans by bites from infected ticks. *Ixodes ricinus* is the main vector for TBEV-EU in Europe, while a role for Dermacentor reticulatus has been suggested [5,6,7]. TBEV-EU infection is often monophasic and starts with an influenza-like prodromal period, followed in 25% of cases by a severe neurological disorder [8,9]. There is no specific treatment and up to 50% of patients who develop neurological complaints and survive TBEV infection can develop long-term sequelae [9]. Despite the availability of a TBEV vaccine, clinical TBEV infection can still occur in vaccinated people [10]. TBEV infections are typically not diagnosed based on a direct detection of TBEV RNA in blood samples, as the viremic phase occurs at the very beginning of disease when symptoms are non-specific and mild. Sampling typically takes place when there are more severe disease manifestations when the patient is in the second phase. Therefore, TBE diagnosis is typically based on the detection of specific antibodies directed against TBEV in the serum or in cerebrospinal fluid of infected individuals [11]. 

TBEV belongs to the orthoflavivirus genus within the Flaviviridae family, which includes several other human pathogens such as West Nile virus (WNV), dengue virus (DENV), Zika virus (ZIKV), Japanese encephalitis virus (JEV) and Usutu virus (USUV) [8]. Three genetic subtypes of TBEV are described, the European (TBEV-Eu), Siberian (TBEV-Sib) and Far Eastern (TBEV-FE) subtypes [8], and two others have been recently proposed: Baikalian (TBEV-Bkl) and Himalayan (Him-TBEV) [12,13]. The molecular organization of orthoflaviviruses is conserved. They are enveloped viruses containing a positive-sense RNA genome of approximately 11,000 bases in length. The genome encodes a single polyprotein cleaved by cellular and viral proteases into three structural proteins: the capsid (C), the precursor membrane (prM) and the envelope (E) proteins; and seven non-structural proteins (NS): NS1, NS2a, NS2b, NS3, NS4a, NS4b and NS5 [14]. NS1 is a dimer protein, with a molecular weight between 46 and 55 kDa depending on its glycosylation pattern [15,16]. NS1 plays a role in viral replication and immune evasion, and can be used as an antigen for the diagnosis of orthoflavivirus infections in serological assays [17,18,19,20]. The orthoflavivirus E protein is organized in three domains: EDI, EDII and EDIII [21,22,23,24]. EDIII has been proposed to be the receptor-binding domain (RBD) and undergoes important repositioning during virus–membrane fusion [21,22]. The protruding position of EDIII suggests that it can be a potent antigen for a serological diagnosis of TBEV infection. Indeed, several studies have shown that EDIII is a major target for neutralizing antibodies (NAbs) [25,26,27,28,29,30] and that it contains virus-specific epitopes [31,32].

The standard test for TBEV-antibody detection, such as an enzyme-linked immunosorbent assay (ELISA) and immunofluorescence assay (IFA), typically use the whole virus or the complete E protein as antigens [33]. However, the detection of TBEV-specific antibodies can be biased by the high level of cross-reactivity among related orthoflavivirus species, as it has been revealed in comparative studies on the commercially available diagnostic kits for TBE infection [33,34]. For instance, false WNV positive results may be generated with the sera of TBEV-infected subjects [35]. In addition, because the circulation of orthoflaviviruses can overlap temporally and spatially, the history of the patient regarding travel, previous infections and vaccinations should be considered when interpreting orthoflavivirus serological assays. 

In previous studies, we have used recombinant NS1 (rNS1) proteins to detect anti-orthoflavivirus IgG in a multiplex NS1-based protein microarray [36,37,38]. The multiplex microarray approach allows a rapid, high-throughput simultaneous detection of antibodies against a number of viral proteins using a low specimen volume (e.g., 10 µL of serum). Here, we have sought to expand our NS1 IgG orthoflavivirus array for the specific and sensitive detection of TBEV IgG elicited by either natural infection or vaccination. To this end, we explored the added value of the reported high sensitivity and specificity of domain III of the E protein (EDIII) [39,40,41]. We could successfully show that the addition of flavivirus EDIII next to the routinely used antigen NS1 increases the sensitivity for TBEV IgG detection. The multiplex assay developed in this study would be a powerful single diagnostic tool to screen for orthoflavivirus infections in endemic regions. 

## 2. Materials and Methods

### 2.1. Cloning and Expression

**Figure 1:**
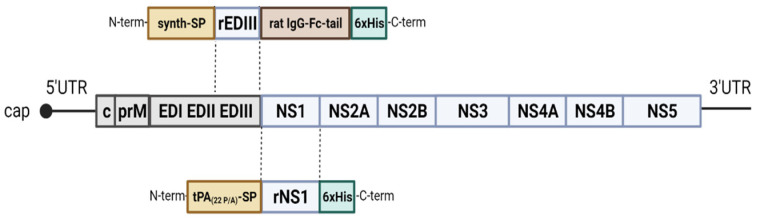
Orthoflavivirus genome organization. Schematic representation of expression vector pPPI4-tPA-(22 P/A)-SP-rNS1-6xHis (lower) and vector pPPI4-synth-SP-rEDIII-Fc-6xHis (upper). The tick-borne encephalitis virus (TBEV) genome encodes 3 structural proteins, the capsid (C), the precursor membrane (prM) and the envelope (E) proteins, and seven non-structural proteins (NS). tPA(22 P/A)-SP corresponds to the tPA signal secretion sequence mutated at position 22 and presents at the N-terminus of the protein. rNS1, recombinant nonstructural protein 1, and 6xHis is the tag at the C-terminus used for protein purification. Synth-SP corresponds to the synthetic signal secretion sequence, at the N-terminus of the protein; rEDIII, recombinant envelop domain-III protein; and Fc, rat IgG-Fc-tail.

TBEV (GenBank acc. nr. AEP25267.2), WNV lineage 2 (AY532665.1), DENV serotype 1 (DENV_1_) (T279761.2), DENV serotype 2 (DENV_2_) (AII99332.1), DENV serotype 3 (DENV_3_) (ALS05358.1), DENV serotype 4 (DENV_4_) (ANC57613.1), ZIKV (MF438286.1), JEV (NP_059434.1) and USUV (AQM55263.1) NS1 synthetic genes (Baseclear, Leinden, The Netherlands) were codon-optimized for expression in human cells and sub-cloned with a Gibson Assembly kit (NEB, Boston, MA) in the high-level mammalian expression vector pPPI4 presenting a hexahistidine (his) tag [42] (i.e., pPPI4-tPA(22 P/A)-SP-rNS1-6xHis and verified via Sanger sequencing. The NS1 genes were preceded by the signal secretion sequence tPA (tPA-SP) mutated at position 22 (22 P/A) p to improve the secretion of the recombinant proteins [43] (Figure 1). The codon-optimized DNA encoding the EDIIIs of TBEV, WNV, DENV_1–4_, ZIKV, JEV and USUV flanked with a synthetic signal secretion sequence (Genscript Biotech, Netherlands) and the sequence coding for a rat IgG-Fc-tail were synthesized and sub-cloned in the pPPI4 expression vector by GenScript (i.e., pPPI4-synth-SP-rEDIII-Fc-6xHis) (Figure 1). All the recombinant proteins were produced in human embryonic kidney (HEK) 293F suspension cells maintained in FreeStyle™ 293 Expression Medium (Gibco, Thermo Fisher Scientific, Breda, The Netherlands), in a shaking incubator (125 r.p.m.) at 37 °C and 8% CO_2_. The pPPI4 orthoflavivirus plasmids (125 µg) were transfected into HEK-293F cells using transfection-grade linear Polyethylenimine Hydrochloride “Max” (PEI MAX) (Polysciences, Bergstrasse, Germany) following the manufacturer’s recommendations. Cells were discarded 7 days post transfection via centrifugation at 4000 r.p.m. for 30 min at 4 °C. The secreted proteins were subsequently purified from the supernatant.

### 2.2. Purification of the Recombinant Proteins

**Figure 2:**
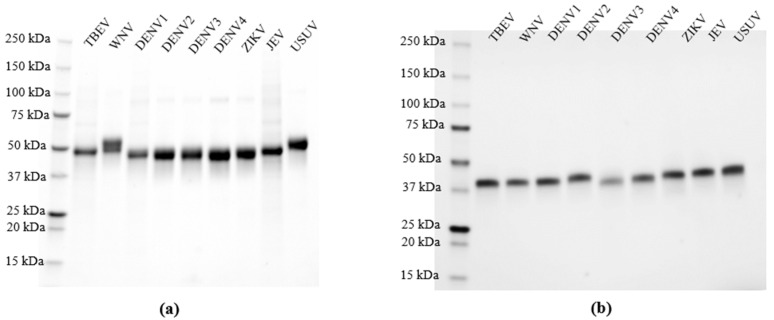
Production of orthoflavivirus recombinant NS1 for sensitive and specific microarray detection of orthoflavivirus IgG. (**a**) Recombinant NS1 and (**b**) recombinant EDIII of TBEV, West Nile virus (WNV) lineage 2, dengue virus (DENV) DENV_1_, DENV_2_, DENV_3_, DENV_4_, Zika virus (ZIKV), Japanese encephalitis virus (JEV) and Usutu virus (USUV) were purified and then analyzed via SDS-PAGE.

The clarified supernatant was incubated with Ni-NTA resin (Bio-Rad, Veenendaal, The Netherlands) at 4 °C for 1 h, with gentle shaking. Beads were transferred to 25 mL cartridges and washed with 20 mL of washing buffer (50 mM Tris (pH 8), 300 mM NaCl and 30 mM imidazole). Recombinant proteins were eluted in 50 mM Tris (pH 8), 300 mM NaCl and 300 mM imidazole. The imidazole was removed via dialysis using a standard grade regenerated cellulose (Spectrum Chemical, Gardena, CA, USA) placed in 2 L of phosphate-buffered saline (PBS) overnight at 4 °C. Finally, proteins were concentrated on Amicon ultrafilters (EMD Millipore, Darmstadt, Germany), and the final concentration was determined using absorbance measurements at 280 nm using a Nanodrop. The purified recombinant proteins were analyzed via sodium dodecyl sulphate polyacrylamide gel electrophoresis (SDS-PAGE), and the purity estimated was higher than 95% (Figure 2a,b). Proteins were stored at −80 °C prior to their utilization.

### 2.3. Serum Samples for Multiplex Protein Microarray Validation 

For the validation of the protein microarray, sera from anonymized patients were used. Table 1 contains the information of each serum cohort. Patients were diagnosed according to internationally accepted criteria, combining clinical symptoms, epidemiological data and outcomes of routine serological methods (ELISA, IFA) [44]. DENV serum samples (*n* = 11) presented IgG against DENV without serotype specification. DENV sera were collected from returning travelers in the Netherlands between 2019 and 2020 and represent individual patients. ZIKV serum samples (*n* = 15) were collected in 2017 and 2020. Two sera were collected in Aruba and two in Bonaire. The remaining samples were collected in the Netherlands from returning travelers. All samples are individual serum samples. As DENV and ZIKV PRNT or VNT were not available, the routine diagnostic outcomes could be confirmed through PRNT or VNT only for the WNV and TBEV serum samples. Seven WNV serum samples representing individual patients were collected in 2020 in the Netherlands. TBEV sera (*n* = 20) were collected in 2008–2021 in the Netherlands. Fourteen samples were from individual patients, while for three patients, paired serum samples were available. For serum samples for which the date of onset of illness was known, the sera were taken >3 weeks post onset. In general, in the Netherlands, diagnostics for TBEV infection are requested late in the course of disease when neurological complaints have manifested and common etiology has been excluded. Serum samples from TBEV-vaccinated patients (*n* = 13) were collected in the Netherlands between 2007 and 2021 and represent individual patients. These patients indicated not to have received a previous YFV and/or JEV vaccination. All vaccinated serum samples were tested TBEV IgG positive and one sample presents TBEV IgM and IgG. While the vaccination statuses of the WNV, DENV and ZIKV patients were not available, the immunization statuses of 11 of 20 TBEV patients were known, of which one reported a previous yellow fever vaccination. Overall, for only ten patients, the date of onset of symptoms had been documented and the time between this date and the date of sample collection varied from five days to four months). The negative control serum cohort (*n* = 35) was collected in the Netherlands (*n* = 30) and in the Caribbean Netherlands (*n* = 5) between 2020 and 2023 and tested negative with routine orthoflavivirus diagnostics.

The sera from 556 nature management workers without TBEV vaccination were collected in the “Dutch forestry study”, as described elsewhere [45]. This study assessed the exposure to TBEV among professionals with a high risk for tick bites in the Netherlands.

The current study was performed in accordance with the guidelines for the sharing of anonymous sera and patient data in observational scientific research in emergency situations as issued by the Commission on the Codes of Conduct of the Federation of Dutch Medical Scientific Societies. The sera of the Dutch forestry workers study were collected under approval # 16–767/D of the medical ethics committee of the University Medical Center in Utrecht, The Netherlands.

### 2.4. Multiplex Protein Microarray

Array slides were produced and treated as described previously [46,47]. TBEV rEDIII and the rNS1 of DENV_1_, DENV_2_, DENV_3_, DENV_4_, JEV, TBEV, USUV, WNV lineage 2 and ZIKV at concentrations of 1.5 mg/mL were mixed with 2× protein printing buffer (GVS, Sandford, FL, USA) and spotted in duplicate in three drops of 333 pL each on 24-pad nitrocellulose-coated slides (ONCYTE AVID, GraceBio Labs, Bend, OR, USA) by using a non-contact Marathon Arrayjet microarray spotter (Roslin, UK). After printing, slides were placed in a drying chamber overnight and stored at room temperature until further use. To avoid non-specific binding to the nitrocellulose surface, the printed slides were treated with Blotto-blocking buffer (ThermoFisher, Breda, The Netherlands) for 1 h at 37 °C in a moist chamber. In between steps, slides were washed 3 times with 1× PBS supplemented with 0.1% Tween. Sera were diluted in Blotto-blocking buffer containing 0.1% Surfact-Amps20 (ThermoFischer, Breda, The Netherlands), and tested in 3-fold dilutions ranging from 1:20 to 1:43,740. After the washing step, the slides were incubated with goat anti-human IgG, F(ab’)2 fragment specific, Alexa Fluor 647-conjugated (Jackson Immuno Research, West Grove, PA, USA), diluted 1:1000 in Blotto-blocking buffer with 0.1% Surfact-Amps20 and incubated for 1 h at 37 °C in a moist chamber. Finally, the slides were washed with sterile water and dried. Signals were quantified using a Power scanner (Tecan, Männedorf, Switzerland).

### 2.5. Data Analysis

A data analysis was performed as previously described [46]. To read the csv files containing the raw data generated by ScanArray Express software version 4.0.0.0004 (PerkinElmer, Waltham, MA, USA), we used R studio v4.0.0, package “DRC” version 2.3-7 (R studio, Boston, MA, USA). A representative theoretical IgG titer (EC50) was calculated based on serial serum dilutions exactly as described previously [48]. For the first screen of samples from the “Dutch forestry study”, based on a single samples dilution (1/20), the analysis of median spot fluorescence foreground intensity determined using ScanArray Express (version 4.0) classified the samples in three groups: (i) samples presenting a saturation intensity signal (intensity > 60,000), (ii) samples presenting a sub-saturation intensity signal (intensity between 40,000 and 60,000) and (iii) samples presenting a low intensity signal (below 40,000). Sera were considered positive for TBEV-specific IgG when above background titers (≤10) for rNS1 and/or rEDIII were detected. 

### 2.6. Plaque Reduction Neutralization Test (PRNT)

The PRNT was used as a confirmatory assay for orthoflavivirus infection. The European subtype of TBEV (TBEV-Eu, strain Salland) and WNV lineage 2 (B956 strain) were used in the PRNT. A549 cells (CCL-185™) were maintained in Dulbecco’s Modified Eagle Medium (DMEM) supplemented with 10% fetal bovine serum (FBS) and 100 U/mL of penicillin–streptomycin. For the assay, cells were seeded at a density of 2.5 × 10 cells/well one day prior to infection. The assay was performed in duplicate using CELLSTAR 24-well cell culture multi-well plates (Greiner Bio-One, Frickenhausen, Germany) in a biosafety level 3 facility (BSL3). Serological specimens were diluted 1:8 in DMEM supplemented with 2% FBS and antibiotic. All diluted sera were heat-inactivated (56 °C, 30 min) before testing. In a 96-well plate, the sera were further diluted 2-fold from 1:8 to 1:2048 in DMEM supplemented with 2% FBS and antibiotic in a volume of 120 μL. Next, virus suspension was mixed to each serum dilution and incubated at 37 °C for 1 h. The virus–serum mixtures were added onto pre-formed A549 cell monolayers and incubated for 1 h at 37 °C in a 5% CO_2_ incubator. The cell monolayer was then overlaid with 3.2% carboxymethyl cellulose (CMC) medium (Acros Organics, Landsmeer, The Netherlands) in a cell culture medium (DMEM containing 2% FBS and antibiotic) and incubated at 37 °C in a 5% CO_2_ incubator. After a 4-day incubation period, the cells were fixed by adding 10% formaldehyde in PBS and stained using 1% crystal (Acros Organics, Landsmeer, The Netherlands) violet in 20% ethanol. Plaques were counted, and the endpoint titers were expressed as reciprocal of the highest serum dilution showing a ≥50% reduction in plaque counts compared to wells without serum. 

### 2.7. Statistical Analysis

Data visualization and statistical analyses were performed in GraphPad Prism software (version 9.1.0). A non-parametric *t*-test was performed to assess the statistical differences of paired or unpaired samples. Significance was noted as ****, *p* < 0.0001; ***, *p* < 0.001; **, *p* < 0.01; *, *p* < 0.05; ns, not significant. The receiving operating characterization (ROC) curve was created by plotting the TBEV seropositive samples confirmed using the PRNT assay against the overall orthoflavivirus seronegative samples. To describe the performance of the TBEV rEDIII as a potent antigen to characterize TBEV serostatus, the area under the ROC curve was calculated as a single numerical measurement. 

## 3. Results

### 3.1. Expression and Validation of Recombinant NS1 for Specific Orthoflavivirus IgG Detection Using Multiplex Protein Microarray 

**Figure 3:**
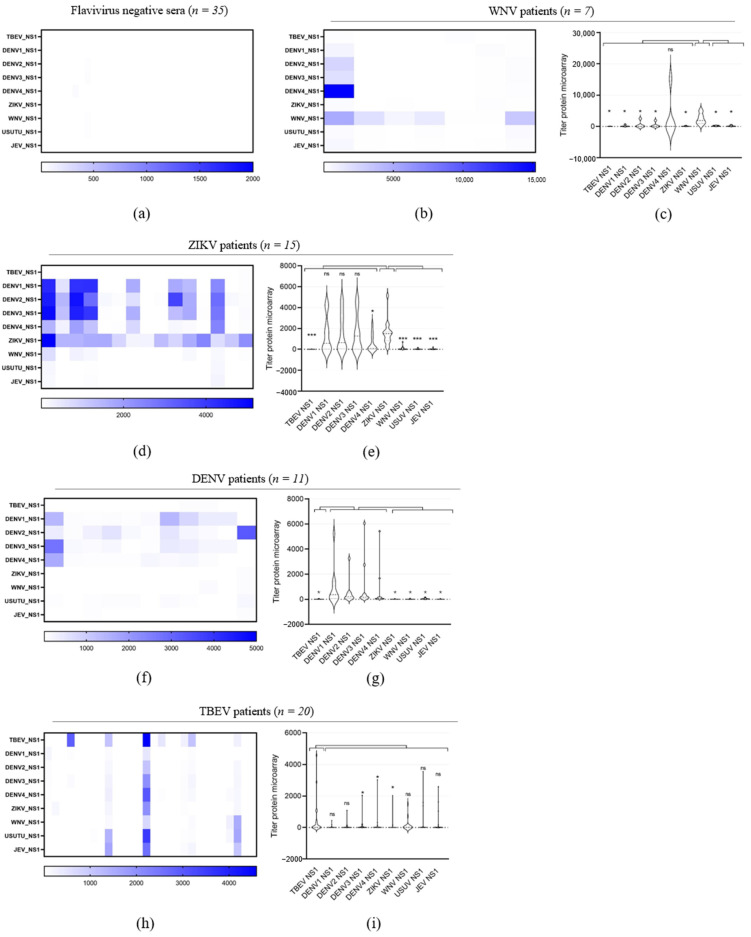
Validation of orthoflavivirus recombinant NS1 for sensitive and specific microarray detection of orthoflavivirus IgG. Heatmaps displaying microarray titers for the orthoflavivirus rNS1 of (**a**) orthoflavivirus negative reference sera, (**b**) WNV patients, (**d**) ZIKV patients, (**f**) DENV patients and (**h**) TBEV patients. (The color key of the titers is indicated at the bottom of each heatmap). The microarray antigens are listed on the left of each heatmap. Violin plots showing microarray titers of (**c**) WNV patients, (**e**) ZIKV patients, (**g**) DENV patients and (**i**) TBEV natural infection-positive sera against the recombinant NS1 of TBEV, DENV1, DENV2, DENV3, DENV4, ZIKV, WNV, USUV and JEV. A non-parametric *t*-test was performed to assess the statistical differences of paired samples. Significance is noted as ***, *p* < 0.001; *, *p* < 0.5. (**b**) Receiving operating characterization (ROC) curve was created with GraphPad Prism software (version 9.1.0).

To improve the yield of NS1 expression in comparison to our previous system [36] and increase flexibility for novel constructs, we changed the pPPI4 eukaryotic expression vector to express our antigen panel of nine NS1 proteins from DENV_1–4_, JEV, TBEV, USUV, WNV and ZIKV. This system yielded up to 10 mg of purified protein per liter of culture with a purity of >95% (Figure 2a). The performance of the array was assessed using a panel of sera from patients with a probable or confirmed [44] ZIKV (*n* = 15), DENV (*n* = 11), WNV (*n* = 7) or TBEV (*n* = 20) infection, confirmed negative for these viruses (*n* = 35) based on routine diagnostic tests, and people who received TBEV vaccination (*n* = 13) (Table 1). Figure 3a–e show heatmaps of the calculated IgG titers of the serum cohorts for each antigen, with columns representing individual sera reactivity to all tested NS1 antigens. All negative control sera showed a negative or near negative IgG titer against the rNS1 (background level) (Figure 3a), indicating an absence of non-specific binding. Three of seven sera reported positive for WNV IgG through routine diagnostics, showing IgG responses exclusively against the WNV rNS1; one of seven reacted with both WNV rNS1 and USUV, JEV and DENV_1–4_ rNS1s, with the highest IgG titer for DENV_4_ rNS1; and one of seven presented, in addition to WNV NS1 reactivity, a low, but above background, reactivity for USUV NS1. Two sera did not bind to any of the NS1 antigens at all (Figure 3b,c). All 15 pre-characterized ZIKV IgG positive sera showed an IgG titer against ZIKV rNS1. In addition, a high-level IgG reactivity to DENV_1–4_ rNS1 antigens was detected for eight of them, and for five of those, a low IgG titer against WNV, JEV and/or USUV NS1 was detected as well. Four of those presented a high IgG titer against DENV_1–4_ rNS1, and one presented a higher titer for DENV_1_, DENV_2_ and DENV_3_ compared to ZIKV NS1 reactivity. Finally, four samples presented a low reactivity to only DENV_2_ with two being close to background level (Figure 3d,e). All DENV control sera showed a titer for at least one of the DENV serotypes, rNS1, while six samples presented varying IgG titers for all serotypes. Two samples showed a titer for DENV_2_ NS1 only, one sample for DENV_3_ only, one sample for both DENV1 and DENV3 and one sample for both DENV_2_ and DENV_3_ (Figure 3f,g). In addition, two samples also presented an IgG titer just over the background level to ZIKV rNS1. Finally, four samples showed a reactivity for TBEV rNS1 (*n* = 2) or ZIKV NS1 (*n* = 2) at the background level (Figure 3f,g). Only 7 of the 20 pre-characterized sera in the TBEV-infection cohort showed IgG reactivity to TBEV rNS1, which included 6 sera corresponding to the three serum pairs in this cohort. The sera from the three serum pairs were taken at least 3 weeks since the onset of illness and 3–5 weeks apart, and showed an increase in rNS1 reactivity in time. Four of the seven reactive sera showed additional IgG reactivity to other rNS1 antigens. In these four cases, two presented a highest titer for TBEV rNS1 while two sera presented a higher titer against JEV, USUV and/or WNV rNS1 (Figure 3h,i). In total, 2 sera reacted with very low titers to non-TBEV rNS1 only, while 11 sera did not react at all. 

To further investigate the observed discrepancies in results with the WNV and TBEV cohorts between our microarray and routine diagnostic testing for WNV and TBEV rNS1, we further characterized specimens using plaque reduction neutralization (PRNT) assays, the gold standard used to confirm orthoflavivirus infection based on serology. 

### 3.2. WNV and TBEV Plaque Reduction Neutralization Test 

To understand the lack of reactivity for WNV rNS1 in the microarray of the two specimens from probable WNV patients, we characterized the complete WNV serum panel in a WNV-specific PRNT using WNV lineage 2. While the five WNV rNS1 reactive WNV patient samples were all able to neutralize WNV, the ones that did not show an IgG titer against WNV rNS1in the array (Figure 3b and Table 1) did not neutralize WNV in the PRNT either, confirming the reactivity measured in the protein microarray.

To investigate the discrepant results among the 20 TBEV patient sera in the rNS1 microarray, a TBEV PRNT was performed (Table 1). Among the 20 probable TBEV infections, the TBEV-neutralizing antibody positivity was 75% (15/20) (titer ≥ 1:16), including the 7 sera confirmed via the rNS1-based microarray. Eight sera were confirmed through the TBEV PRNT but not via rNS1 microarray, which is indicative of a lack of sensitivity of the protein microarray using NS1-based antigens. 

### 3.3. Evaluation of TBEV EDIII as Antigen for TBEV IgG Detection

**Figure 4:**
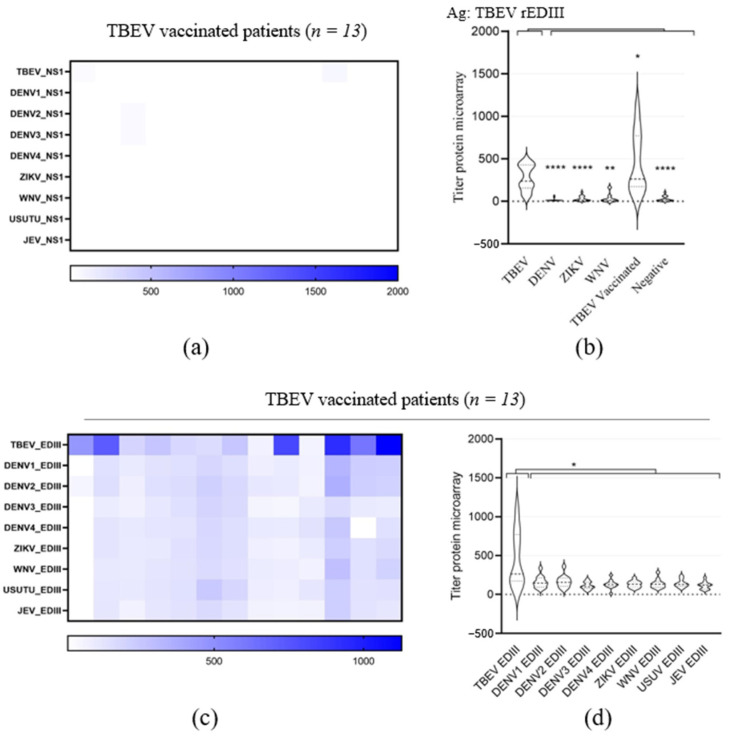
(**a**) Heatmap displaying microarray titers for the orthoflavivirus rNS1 of orthoflavivirus TBEV-vaccinated patients. (The color key of the titers is indicated at the bottom of the heatmap). The microarray antigens are listed on the left of the heatmap. (**b**) Violin plot showing microarray titers of the pre-characterized TBEV, dengue virus (DENV), Zika virus (ZIKV), West Nile virus (WNV) sera and the negative orthoflavivirus sera against TBEV rEDIII. TBEV vaccinees were used as the positive control. (**c**) Heatmap displaying microarray titers for the orthoflavivirus rEDIII of orthoflavivirus TBEV-vaccinated patients. (The color key of the titers indicated at the bottom of the heatmap.) The microarray antigens are listed on the y-axis. (**d**)Violin plot showing microarray titers of TBEV-vaccinated serum samples against the recombinant EDIII of TBEV, DENV1, DENV2, DENV3, DENV4, ZIKV, WNV, USUV and JEV. A non-parametric T-test was performed to assess the statistical differences of paired samples. Significance is noted as ****, *p* < 0.0001; **, *p* < 0.01; *, *p* < 0.5. The order of patient samples is the same for each heatmap.

To improve the sensitivity for TBEV IgG detection while maintaining or even improving the high level of specificity, we evaluated the parallel use of the orthoflavivirus antigen EDIII in the microarray. TBEV, DENV1–4, ZIKV, WNV, USUV and JEV rEDIII were expressed in mammalian cells as secreted proteins (Figure 1b). As available TBEV vaccines do not lead to the production of NS1 in vaccinees but do elicit E protein-directed antibodies, the sera of people vaccinated against TBEV can be used as a positive control for TBEV rEDIII. As expected, these serum samples (*n* = 13) presented IgG reactivity for TBEV rEDIII, whereas no or only background IgG reactivity against TBEV rNS1 was detected (Figure 4). The microarray with TBEV rEDIII demonstrated a specific reaction against TBEV infection and vaccination sera versus WNV, DENV and ZIKV sera and the negative cohort (Figure 4b). While only 7 of the 20 sera of probable TBEV patients showed reactivity against TBEV rNS1 (Figure 3h,i), 15 of these presented IgG reactivity to TBEV rEDIII antigen. This was completely consistent with the PRNT results (Table 1). The samples that were not able to neutralize TBEV did not show any reactivity against rEDIII either. The comparison of PRNT and microarray results shows that low microarray titers nearly always correspond to a low or a lack of neutralization potency. Only IgG titers against rEDIII > 100 on microarray correspond to measurable neutralization titers, as sera that presented negative on the PRNT showed titers < 100. Lastly, we tested the reactivity of the TBEV vaccinated sera to other orthoflavivirus rEDIII proteins (Figure 4c,d). Only a low level of IgG reactivity was detected against WNV, DENV1–4, ZIKV, USUV and JEV rEDIII, but a much higher titer was found for TBEV rEDIII (Figure 4c,d). So, despite some cross-reactivity, the observed titers clearly indicated TBEV exposure.

**Figure 5:**
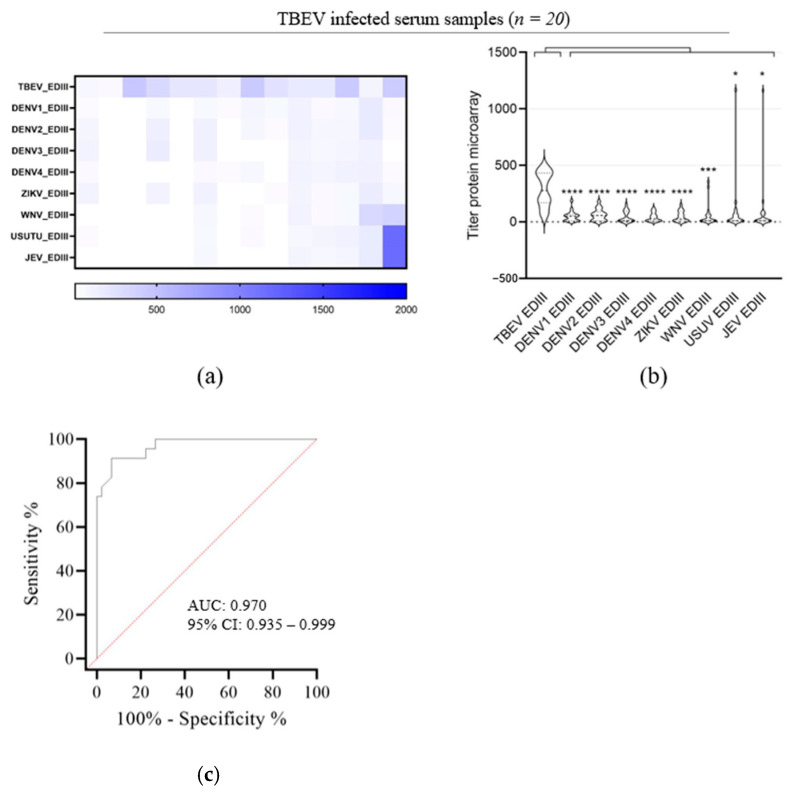
TBEV-infected serum samples against recombinant EDIII. (**a**) Heatmap displaying microarray titers for the orthoflavivirus rEDIII of orthoflavivirus TBEV natural infection-positive sera. (The color key of the titers is indicated at the bottom of each heatmap). The microarray antigens are listed on the y-axis. (**b**) Violin plots showing the microarray titers of TBEV natural infection-positive sera against the recombinant EDIII of TBEV, DENV1, DENV2, DENV3, DENV4, ZIKV, WNV, USUV and JEV. A non-parametric T-test was performed to assess the statistical differences of paired samples. Significance is noted as ****, *p* < 0.0001; ***, *p* < 0.001; *, *p* < 0.5. (**c**) Receiving operating characterization (ROC) curve was created with GraphPad Prism software (version 9.1.0). The order of patient samples is the same as in Figure 3.

Next, we evaluated the reactivity of the TBEV natural infection sera to WNV, DENV1–4, ZIKV, USUV and JEV rEDIII proteins (Figure 5). The samples presented only low levels of IgG reactivity to WNV, DENV1–4, ZIKV, USUV and JEV rEDIII compared to those of IgG reactivity to TBEV rEDIII. Within those samples, some serum samples presented high IgG reactivity to WNV, USUV or JEV rEDIII (Figure 5a,b) as well as IgG reactivity to rNS1 antigen for those orthoflaviviruses (Figure 3h,i), which is most probably indicative for a previous orthoflavivirus infection.

To evaluate the performance of the microarray test with rEDIII as an antigen to detect TBEV infection, a receiving operating characterization (ROC) analysis [49] was performed. The ROC analysis was created by plotting the 15 TBEV seropositive samples confirmed through the PRNT assay against the overall orthoflavivirus seronegative samples. The ROC curve analysis indicates that the protein microarray is accurate in determining the TBEV serostatus relative to the PRNT50 assay results with an area under the curve (AUC) of 0.970 (Figure 5c).

**Figure 6:**
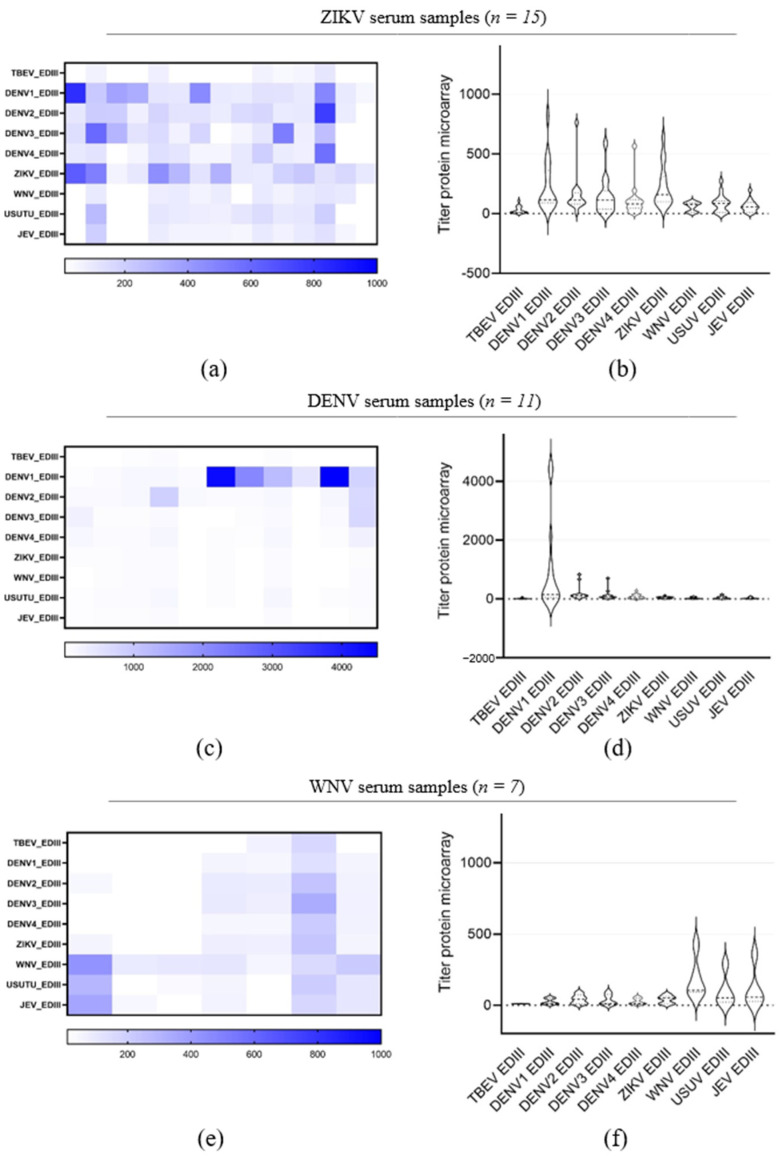
Evaluation of recombinant orthoflavivirus EDIII as an antigen. Heatmaps and violin plots showing the microarray titers of ZIKV-positive sera (**a**,**b**), DENV-positive sera (**c**,**d**) and WNV-positive sera (**e**,**f**) against the recombinant rEDIII of TBEV, DENV1, DENV2, DENV3, DENV4, ZIKV, WNV, USUV and JEV. The order of patient samples is the same as in Figure 3.

We also evaluated the reactivities of the DENV, ZIKV and WNV serum samples against the range of orthoflaviviruses rEDIII antigens (Figure 6). ZIKV serum samples showed an IgG reactivity to all orthoflavivirus rEDIII but with higher titers for DENV1–4 and ZIKV rEDIII (Figure 6a,b). DENV serum samples presented an IgG titer against DENV1 rEDIII only (Figure 6c,d). For sera from positive WNV patients, high-level IgG reactivity against WNV rEDIII and the most closely related JEV and USUV rEDIIIs was detected with the highest titer against WNV rEDIII. In addition, those sera presented IgG titers against DENV1–4, ZIKV and TBEV rEDIII close to the background (Figure 6e,f). 

### 3.4. Performance Assessment of rNS1/rEDIII Microarray for TBEV IgG Detection in High-Risk Cohort of Forestry Workers

Finally, we assessed the performance of the array in a public health application, using the sera collected from 556 participants in the “Dutch forestry workers” study. Participants who reported to have received a TBEV vaccination were excluded [45]. To pre-select TBEV-antigen reactive sera for full titration, all 556 sera were first screened for TBEV antigen reactivity by using a single serum dilution (1/20) against the 18 orthoflavivirus antigens (9 rNS1 and 9 rEDIII). The serum samples (*n* = 5) presenting a specific positive signal for TBEV antigens (rNS1 and/or rEDIII) with little to no cross-reactivity to other orthoflavivirus, were tittered, as previously described. None of the five serum samples showed an IgG titer against TBEV rNS1 (Table 2). Three samples presented a high IgG reactivity to TBEV rEDIII with a titer > 100, and two serum samples had an IgG titer against TBEV rEDIII < 100 but above the background level (Table 2). We then compared these results with the previous results [45]. The five samples that tested TBEV positive (rNS1 and/or rEDIII reactivity) in the microarray were indeed all included in the 10 serum samples that tested positive in the TBEV ELISA while only three of them had tested positive in a TBEV PRNT (Table 2) [45]. The TBEV-neutralizing sera corresponded to the three sera with a titer > 100 against rEDIII on the microarray, thereby confirming our previous assertion that only IgG titers against rEDIII > 100 on the microarray can result in measurable neutralization titers. 

## 4. Discussion

The diagnosis of TBEV infection often relies on serological methods [50,51]. However, orthoflavivirus diagnosis based on serology is highly complex due to the spatiotemporal overlap of the circulation of closely related orthoflaviviruses that elicit cross-reacting antibody responses and have overlapping disease manifestations [51,52]. Comparative virus neutralization assays are the gold standard for a serology-based confirmation of orthoflavivirus infection, but are not routinely performed by diagnostic labs due to complexity and the need for high-containment laboratories. Our multiplex arbovirus approach allows for a rapid, high-throughput simultaneous detection of antibodies against a number of orthoflavivirus proteins using a low specimen volume (e.g., 10 µL). We have sought to expand our NS1 IgG orthoflavivirus array for the specific and sensitive detection of TBEV IgG elicited by either natural infection or vaccination. To this emd, we explored the added value of the reported high sensitivity and specificity of the domain III of the E protein (EDIII) [25,26,27,28,29,30,31,53]. We showed that the addition of orthoflavivirus EDIII next to the routinely used antigen NS1 increased the microarray sensitivity for TBEV IgG detection and enabled us to set a microarray titer cut-off (titer 100) that is predictive for positivity in the TBEV PRNT. While only 7 of 15 TBEV PRNT-positive sera showed reactivity against TBEV rNS1, all 15 sera were reactive with TBEV rEDIII on the microarray. Sera that were TBEV IgG positive in routine diagnostic tests but negative for TBEV rNS1 and rEDIII in the microarray could not neutralize TBEV. We cannot 100% exclude that some of the sera in the TBEV-infection cohort were taken very early in the course of the disease when the NS1 IgG responses have not yet appeared [54,55]. As TBEV-neutralizing antibody responses are mainly targeting the E protein, this might result in a positive neutralization test, a negative rNS1 array and a positive array EDIII outcome. However, TBEV infections are typically being diagnosed very late in the course of infection in the Netherlands, when more common etiology has been excluded and when NS1 IgG responses can be expected to be abundant.

Holbrook et al. described results for both the sensitive and specific detection of TBEV IgG using rEDIII. They observed that rabbit antisera raised against TBEV rEDIII protein reacted specifically with TBEV EDIII but not with rEDIII from other orthoflaviviruses such as DENV, WNV and JEV, similar to our observations with TBEV infection and vaccination sera [31] (Figure 4c and Figure 5). A study by the same authors with WNV rEDIII showed some cross-reactivity of rabbit anti-WNV rEDIII serum with rEDIII from viruses in the same JEV-serocomplex [32]. In line, our data show a similar low cross-reactivity of WNV serum samples with the rEDIII of the two other JEV serocomplex viruses, USUV and JEV (Figure 6e). JEV is a travel-related infection that is rarely imported to Europe, while USUV has low endemic circulation in birds in the Netherlands and human infections are rare [56,57,58,59]. It is therefore unlikely that the observed heterologous reactivity is due to past infections. When compared to the rEDIII, our results show a higher specificity of rNS1 for the detection of WNV infection. This is in line with previous observations by Natalie et al. [36]. The detected reactivity with DENV rNS1 can most probably be explained by a travel-related previous infection with DENV. After malaria, dengue is the second-most diagnosed illness in travelers [60].

Previous studies have shown that ZIKV rEDIII is a useful antigen to discriminate between ZIKV and DENV infections [39,40,61]. We, however, observed clear reactivity (but less than that for the homologous antigen–antisera combination) of ZIKV sera with the rEDIII of the four DENV serotypes but not vice versa (Figure 6a,b). The absence of cross-reactivity between DENV sera and ZIKV rEDIII was also observed by Premkumar et al. for late convalescent sera. However, they did observe some cross-reactivity with DENV sera taken in early convalescence [41]. It is, however, difficult to discern between cross-reactivity and past infections in the observed reactivity of ZIKV sera with DENV rEDIII. Within the orthoflavivirus genus, DENV and ZIKV are phylogenetically closely related but ZIKV and DENV are also co-circulating in large parts of the world [62]. Dengue is a regularly diagnosed illness in travelers [60], and the reactivity of the sera in our ZIKV cohort with DENV rEDIII could very well reflect a previous infection in the returning traveler with an acute ZIKV infection. The presence of DENV IgG in these specific samples of our ZIKV serum cohort was also observed in the rNS1-based microarray (Figure 3d). 

Previous epitope analyses of both DENV E protein and NS1 indicated that NS1 could be more useful for specific serology as NS1 contains more virus-specific epitopes [63,64,65,66,67]. Indeed, while the DENV serum cohort presented varying anti-rNS1 IgG titers against the four DENV serotypes, the sera reacted only with DENV1 rEDIII. To correctly evaluate the use of rNS1 versus rEDIII in the serotyping of DENV infection, a serum cohort of serotyped DENV infections will be needed.

Serological discrimination between TBEV infection and vaccination is important to diagnose and study vaccine breakthrough cases of infection [68]. Multiple studies demonstrated that the detection of NS1-specific antibodies could be a tool to distinguish TBEV-specific antibody responses elicited by infection from those elicited by vaccination based on inactivate viruses [69,70,71,72]. Nevertheless, anti-NS1 IgG responses were measured in some vaccinees with a presumed absence of history of TBEV infection [70,71] possibly due to the presence of trace amounts of NS1 protein in preparations of the two TBEV vaccines marketed in Europe [73]. We observed no rNS1 antigen responses in our cohort of vaccinees but were surprised by the absence of rNS1 reactivity in the majority (13 of 15) of our PRNT-confirmed infection sera (Figure 3h and Figure 4a). Although this lack of sensitivity could be overcome by the addition of rEDIII to the microarray analysis (Figure 5), we cannot completely exclude that 5 sera of the 13 infection sera that did not react with rNS1 were not related to TBEV vaccination. Due to privacy regulations, we could not relate back to detailed anamnestic data for those sera. Although uncertainties with respect to the infection versus vaccination status of these five sera exist, the well-defined cohort of confirmed non-vaccinated workers in professions with high risk for tick exposure confirmed the observed higher sensitivity of rEDIII versus rNS1 in the detection of TBEV infections [45].

Our results with the confirmed non-vaccinated high-risk tick exposure study cohort and the combined rNS1/rEDIII microarray are consistent with earlier analyses on this cohort [45]. The sera of three workers that were positive for TBEV infection in the routine ELISA and in the PRNT in the study showed anti-rEDIII IgG reactivity but no anti-rNS1 reactivity on our microarray. Besides the observations in some studies that the currently employed TBEV vaccines might elicit low-level anti-NS1 immune responses and thereby hamper a serology-based distinction between vaccine- and infection-related induced immunity, the data here demonstrate that the absence of NS1-induced immune responses in combination with the presence of EDIII-induced responses does not identify vaccination cases with 100% certainty.

To be more conclusive in discriminating vaccine-induced and infection-induced immune responses, a better understanding of the kinetics of those immune responses in vaccination and infection cohorts with different clinical courses is needed for a more precise interpretation of the data. In conclusion, we showed that for orthoflavivirus IgG serology, with a focus on TBEV, a combined analysis of rEDIII- and rNS1-induced immunity has the potential to provide sensitive and specific data. 

## Figures and Tables

**Table 1:** Overview of serum collection used for orthoflavivirus microarray validation.

Virus Species	Number of Samples	Sex	Group Age (in Years)	Positive Serology (ELISA/IFA) Specific IgG	Virus Neutralization Confirmed (VNT/PRTN)	Vaccination
DENV_1–4_	11	M (6/11) F (5/11)	19 to 70	11/11	N/D	N/A
ZIKV	15	M (3/15) F (12/15)	26 to 80	15/15 (CHIKV and DENV positive IgG IFA: 7/15)	N/D	N/A
WNV	7	M (3/7) F (4/7)	30 to 75	7/7	5/7	N/A
TBEV	20	M (9/20) F (8/20)	20 to 80	20/20	15/20	9/20 N/A 10/20 No vaccine 1/20 YF vaccine
TBEV Vaccinated group	13	M (4/13) F (9/13)	17 to 70	13/13	N/D	TBEV vaccine *
Negative group	35	N/A	N/A	Orthoflavivirus IgM/IgG Neg	N/D	N/A
Dutch forestry workers	556	N/A	22 to 88	TBEV IgG (10/556)	TBEV VNT (3/10)	No vaccine

DENV_1–4_: dengue virus serotypes 1 to 4; WNV: West Nile virus; ZIKV: Zika virus; TBEV: tick-borne encephalitis virus; CHIKV: chikungunya virus. Vaccinated group: * TBEV vaccine (FSME-IMMUN^®^, Pfizer). N/A, information not available. N/D, not determined, assay not available. VNT: virus neutralization test. PRNT: plaque reduction neutralization test. M: male; F: female; YF: yellow fever.

**Table 2:** All TBEV serology results of sera (*n* = 5) that tested TBEV IgG positive for rNS1 and/or rEDIII in the orthoflavivirus microarray.

	Microarray Value				
Sample	NS1 TBEV	EDIII TBEV	TBEV ELISA Value *	VNT Titers *	Interpretation
1	≤10	218	6.1	120	Confirmed TBEV infection
2	≤10	126	3.0	30	Confirmed TBEV infection
3	≤10	101	1.3	30	Confirmed TBEV infection
4	≤10	36	2.6	Neg	Possible TBEV infection
5	≤10	40	2.4	Neg	Possible TBEV infection

* From [45].

## Data Availability

The data presented in this study are available on request from the corresponding author.

## References

[1] Bogovic P., Strle F. (2015). Tick-borne encephalitis: A review of epidemiology, clinical characteristics, and management. World J. Clin. Cases.

[2] Beauté J., Spiteri G., Warns-Petit E., Zeller H. (2018). Tick-borne encephalitis in Europe, 2012 to 2016. Euro Surveill. Bull. Eur. Sur Mal. Transm. Eur. Commun. Dis. Bull..

[3] Dekker M., Laverman G.D., de Vries A., Reimerink J., Geeraedts F. (2019). Emergence of tick-borne encephalitis (TBE) in the Netherlands. Ticks Tick-Borne Dis..

[4] Reimerink J., Sprong H., Harms M., Reusken C.B.E.M. (2023). Netherlands, The TBE Book.

[5] Dumpis U., Crook D., Oksi J. (1999). Tick-borne encephalitis. Clin. Infect. Dis. Off. Publ. Infect. Dis. Soc. Am..

[6] Ličková M., Havlíková S.F., Sláviková M., Slovák M., Drexler J.F., Klempa B. (2020). Dermacentor reticulatus is a vector of tick-borne encephalitis virus. Ticks Tick-Borne Dis..

[7] Chitimia-Dobler L., Lemhöfer G., Król N., Bestehorn M., Dobler G., Pfeffer M. (2019). Repeated isolation of tick-borne encephalitis virus from adult Dermacentor reticulatus ticks in an endemic area in Germany. Parasites Vectors.

[8] Lindquist L., Vapalahti O. (2008). Tick-borne encephalitis. Lancet.

[9] Kaiser R. (2008). Tick-Borne Encephalitis. Infect. Dis. Clin. North Am..

[10] Dobler G., Kaier K., Hehn P., Böhmer M., Kreusch T., Borde J. (2020). Tick-borne encephalitis virus vaccination breakthrough infections in Germany: A retrospective analysis from 2001 to 2018. Clin. Microbiol. Infect. Off. Publ. Eur. Soc. Clin. Microbiol. Infect. Dis..

[11] Holzmann H. (2003). Diagnosis of tick-borne encephalitis. Vaccine.

[12] Kovalev S.Y., Mukhacheva T.A. (2017). Reconsidering the classification of tick-borne encephalitis virus within the Siberian subtype gives new insights into its evolutionary history. Infect. Genet. Evol. J. Mol. Epidemiol. Evol. Genet. Infect. Dis..

[13] Dai X., Shang G., Lu S., Yang J., Xu J. (2018). A new subtype of eastern tick-borne encephalitis virus discovered in Qinghai-Tibet Plateau, China. Emerg. Microbes Infect..

[14] Chambers T.J., Hahn C.S., Galler R., Rice C.M. (1990). Flavivirus genome organization, expression, and replication. Annu. Rev. Microbiol..

[15] Winkler G., Randolph V.B., Cleaves G.R., Ryan T.E., Stollar V. (1988). Evidence that the mature form of the flavivirus nonstructural protein NS1 is a dimer. Virology.

[16] Pryor M.J., Wright P.J. (1994). Glycosylation Mutants of Dengue Virus NS1 Protein. J. Gen. Virol..

[17] Alcon S., Talarmin A., Debruyne M., Falconar A., Deubel V., Flamand M. (2002). Enzyme-linked immunosorbent assay specific to dengue virus type 1 nonstructural protein NS1 reveals circulation of the antigen in the blood during the acute phase of disease in patients experiencing primary or secondary infections. J. Clin. Microbiol..

[18] Ding X.-X., Li X.-F., Deng Y.-Q., Guo Y.-H., Hao W., Che X.-Y., Qin C.-F., Fu N. (2014). Development of a double antibody sandwich ELISA for West Nile virus detection using monoclonal antibodies against non-structural protein 1. PLoS ONE.

[19] Kumar J.S., Parida M., Rao P.L. (2011). Monoclonal antibody-based antigen capture immunoassay for detection of circulating non-structural protein NS1: Implications for early diagnosis of japanese encephalitis virus infection. J. Med. Virol..

[20] Mora-Cárdenas E., Aloise C., Faoro V., Gašper N.K., Korva M., Caracciolo I., D’agaro P., Avšič-Županc T., Marcello A. (2020). Comparative specificity and sensitivity of NS1-based serological assays for the detection of flavivirus immune response. PLoS Neglected Trop. Dis..

[21] Rey F.A., Heinz F.X., Mandl C., Kunz C., Harrison S.C. (1995). The envelope glycoprotein from tick-borne encephalitis virus at 2 Å resolution. Nature.

[22] Modis Y., Ogata S., Clements D., Harrison S.C. (2003). A ligand-binding pocket in the dengue virus envelope glycoprotein. Proc. Natl. Acad. Sci. USA.

[23] Modis Y., Ogata S., Clements D., Harrison S.C. (2005). Variable surface epitopes in the crystal structure of dengue virus type 3 envelope glycoprotein. J. Virol..

[24] Nybakken G.E., Nelson C.A., Chen B.R., Diamond M.S., Fremont D.H. (2006). Crystal structure of the West Nile virus envelope glycoprotein. J. Virol..

[25] Wahala W., Kraus A.A., Haymore L.B., Accavitti-Loper M.A., de Silva A.M. (2009). Dengue virus neutralization by human immune sera: Role of envelope protein domain III-reactive antibody. Virology.

[26] Chen H.-W., Liu S.-J., Li Y.-S., Liu H.-H., Tsai J.-P., Chiang C.-Y., Chen M.-Y., Hwang C.-S., Huang C.-C., Hu H.-M. (2013). A consensus envelope protein domain III can induce neutralizing antibody responses against serotype 2 of dengue virus in non-human primates. Arch. Virol..

[27] Yang X., Qi J., Peng R., Dai L., Gould E.A., Gao G.F., Tien P. (2019). Molecular Basis of a Protective/Neutralizing Monoclonal Antibody Targeting Envelope Proteins of both Tick-Borne Encephalitis Virus and Louping Ill Virus. J. Virol..

[28] Tai W., He L., Wang Y., Sun S., Zhao G., Luo C., Li P., Zhao H., Fremont D.H., Li F. (2018). Critical neutralizing fragment of Zika virus EDIII elicits cross-neutralization and protection against divergent Zika viruses. Emerg. Microbes Infect..

[29] Beasley D.W.C., Barrett A.D.T. (2002). Identification of neutralizing epitopes within structural domain III of the West Nile virus envelope protein. J. Virol..

[30] Agudelo M., Palus M., Keeffe J.R., Bianchini F., Svoboda P., Salát J., Peace A., Gazumyan A., Cipolla M., Kapoor T. (2021). Broad and potent neutralizing human antibodies to tick-borne flaviviruses protect mice from disease. J. Exp. Med..

[31] Holbrook M.R., Shope R.E., Barrett A.D.T. (2004). Use of recombinant E protein domain III-based enzyme-linked immunosorbent assays for differentiation of tick-borne encephalitis serocomplex flaviviruses from mosquito-borne flaviviruses. J. Clin. Microbiol..

[32] Beasley D.W.C., Holbrook M.R., da Rosa A.P.A.T., Coffey L., Carrara A.-S., Phillippi-Falkenstein K., Bohm R.P., Ratterree M.S., Lillibridge K.M., Ludwig G.V. (2004). Use of a recombinant envelope protein subunit antigen for specific serological diagnosis of west Nile Virus infection. J. Clin. Microbiol..

[33] Reusken C., Boonstra M., Rugebregt S., Scherbeijn S., Chandler F., Avšič-Županc T., Vapalahti O., Koopmans M., GeurtsvanKessel C.H. (2019). An evaluation of serological methods to diagnose tick-borne encephalitis from serum and cerebrospinal fluid. J. Clin. Virol..

[34] Niedrig M., Vaisviliene D., Teichmann A., Klockmann U., Biel S. (2001). Comparison of six different commercial IgG-ELISA kits for the detection of TBEV-antibodies. J. Clin. Virol. Off. Publ. Pan Am. Soc. Clin. Virol..

[35] Papa A., Karabaxoglou D., Kansouzidou A. (2011). Acute West Nile virus neuroinvasive infections: Cross-reactivity with dengue virus and tick-borne encephalitis virus. J. Med. Virol..

[36] Cleton N., Godeke G.-J.-J., Reimerink J., Beersma M., van Doorn H.R., Franco L., Goeijenbier M., Jimenez-Clavero M.A., Johnson B.W., Niedrig M. (2015). Spot the difference—Development of a syndrome based protein microarray for specific serological detection of multiple flavivirus infections in travelers. PLoS Neglected Trop. Dis..

[37] Cleton N.B., van Maanen K., Bergervoet S.A., Bon N., Beck C., Godeke G.-J., Lecollinet S., Bowen R., Lelli D., Nowotny N. (2017). A Serological Protein Microarray for Detection of Multiple Cross-Reactive Flavivirus Infections in Horses for Veterinary and Public Health Surveillance. Transbound. Emerg. Dis..

[38] Thao T.T.N., de Bruin E., Phuong H.T., Vy N.H.T., Ham H.-J.v.D., Wills B.A., Tien N.T.H., Le Duyen H.T., Trung D.T., Whitehead S.S. (2021). Using NS1 Flavivirus Protein Microarray to Infer Past Infecting Dengue Virus Serotype and Number of Past Dengue Virus Infections in Vietnamese Individuals. J. Infect. Dis..

[39] Ndiaye O., Diagne C.T., El Wahed A.A., Dia F., Dia M., Faye A., Leal S.D.V., dos Santos M., Mendonça M.d.L.d.L., Leite C.C.d.S. (2023). Use of Envelope Domain III Protein for the Detection of IgG Type Antibodies Specific to Zika Virus by Indirect ELISA. Diagnostics.

[40] Denis J., Attoumani S., Gravier P., Tenebray B., Garnier A., Briolant S., de Laval F., Chastres V., Grard G., Leparc-Goffart I. (2019). High specificity and sensitivity of Zika EDIII-based ELISA diagnosis highlighted by a large human reference panel. PLoS Neglected Trop. Dis..

[41] Premkumar L., Collins M., Graham S., Liou G.-J.A., Lopez C.A., Jadi R., Balmaseda A., Brackbill J.A., Dietze R., Camacho E. (2018). Development of Envelope Protein Antigens to Serologically Differentiate Zika Virus Infection from Dengue Virus Infection. J. Clin. Microbiol..

[42] Binley J.M., Sanders R.W., Clas B., Schuelke N., Master A., Guo Y., Kajumo F., Anselma D.J., Maddon P.J., Olson W.C. (2000). A recombinant human immunodeficiency virus type 1 envelope glycoprotein complex stabilized by an intermolecular disulfide bond between the gp120 and gp41 subunits is an antigenic mimic of the trimeric virion-associated structure. J. Virol..

[43] Wang J.-Y., Song W.-T., Li Y., Chen W.-J., Yang D., Zhong G.-C., Zhou H.-Z., Ren C.-Y., Yu H.-T., Ling H. (2011). Improved expression of secretory and trimeric proteins in mammalian cells via the introduction of a new trimer motif and a mutant of the tPA signal sequence. Appl. Microbiol. Biotechnol..

[44] European Commission (2018). Commission Implementing Decision (EU) 2018/945 of 22 June 2018 on the communicable diseases and related special health issues to be covered by epidemiological surveillance as well as relevant case definitions. OJEU.

[45] Hofhuis A., Berg O.v.D., Meerstadt-Rombach F., Wijngaard C.v.D., Chung N., Franz E., Reimerink J. (2021). Exposure to tick-borne encephalitis virus among nature management workers in the Netherlands. Ticks Tick-Borne Dis..

[46] Koopmans M., de Bruin E., Godeke G.-J., Friesema I., van Gageldonk R., Schipper M., Meijer A., van Binnendijk R., Rimmelzwaan G., de Jong M.D. (2012). Profiling of humoral immune responses to influenza viruses by using protein microarray. Clin. Microbiol. Infect. Off. Publ. Eur. Soc. Clin. Microbiol. Infect. Dis..

[47] Reusken C., Mou H., Godeke G.J., van der Hoek L., Meyer B., A Müller M., Haagmans B., de Sousa R., Schuurman N., Dittmer U. (2013). Specific serology for emerging human coronaviruses by protein microarray. Euro Surveill. Bull. Eur. Sur Mal. Transm. Eur. Commun. Dis. Bull..

[48] van Tol S., Mögling R., Li W., Godeke G.-J., Swart A., Bergmans B., Brandenburg A., Kremer K., Murk J.-L., van Beek J. (2020). Accurate serology for SARS-CoV-2 and common human coronaviruses using a multiplex approach. Emerg. Microbes Infect..

[49] Berrar D., Flach P. (2012). Caveats and pitfalls of ROC analysis in clinical microarray research (and how to avoid them). Brief. Bioinform..

[50] Pustijanac E., Buršić M., Talapko J., Škrlec I., Meštrović T., Lišnjić D. (2023). Tick-Borne Encephalitis Virus: A Comprehensive Review of Transmission, Pathogenesis, Epidemiology, Clinical Manifestations, Diagnosis, and Prevention. Microorganisms.

[51] da Silva P.G., dos Reis J.A.S., Rodrigues M.N., Ardaya Q.d.S., Mesquita J.R. (2023). Serological Cross-Reactivity in Zoonotic Flaviviral Infections of Medical Importance. Antibodies.

[52] Makino Y., Tadano M., Saito M., Maneekarn N., Sittisombut N., Sirisanthana V., Poneprasert B., Fukunaga T. (1994). Studies on serological cross-reaction in sequential flavivirus infections. Microbiol. Immunol..

[53] Sánchez M.D., Pierson T.C., DeGrace M.M., Mattei L.M., Hanna S.L., Del Piero F., Doms R.W. (2007). The neutralizing antibody response against West Nile virus in naturally infected horses. Virology.

[54] Svoboda P., Haviernik J., Bednar P., Matkovic M., Rincón T.C., Keeffe J., Palus M., Salat J., Agudelo M., Nussenzweig M.C. (2023). A combination of two resistance mechanisms is critical for tick-borne encephalitis virus escape from a broadly neutralizing human antibody. Cell Rep..

[55] Matveeva V.A., Popova R.V., Kvetkova E.A., Chernicina L.O., Zlobin V.I., Puchovskaya N.M., Morozova O.V. (1995). Antibodies against tick-borne encephalitis virus (TBEV) non-structural and structural proteins in human sera and spinal fluid. Immunol. Lett..

[56] Erlanger T.E., Weiss S., Keiser J., Utzinger J., Wiedenmayer K. (2009). Past, present, and future of japanese encephalitis. Emerg. Infect. Dis..

[57] Platonov A.E., Rossi G., Karan L.S., O Mironov K., Busani L., Rezza G. (2012). Does the Japanese encephalitis virus (JEV) represent a threat for human health in Europe? Detection of JEV RNA sequences in birds collected in Italy. Euro Surveill. Bull. Eur. Sur Mal. Transm. Eur. Commun. Dis. Bull..

[58] Zaaijer H.L., Slot E., Molier M., Reusken C.B., Koppelman M.H. (2019). Usutu virus infection in Dutch blood donors. Transfusion.

[59] Rijks J.M., Kik M.L., Slaterus R., Foppen R., Stroo A., Ijzer J., Stahl J., Gröne A., Koopmans M., van der Jeugd H.P. (2016). Widespread Usutu virus outbreak in birds in the Netherlands, 2016. Eurosurveillance.

[60] Grobusch M.P., Weld L., Goorhuis A., Hamer D.H., Schunk M., Jordan S., Mockenhaupt F.P., Chappuis F., Asgeirsson H., Caumes E. (2021). Travel-related infections presenting in Europe: A 20-year analysis of EuroTravNet surveillance data. Lancet Reg. Health Eur..

[61] Prates J.W.O., Xisto M.F., Rodrigues J.V.d.S., Colombari J.P.C., Meira J.M.A., Dias R.S., da Silva C.C., de Paula E.S.O. (2022). Zika Virus Envelope Protein Domain III Produced in *K. phaffii* Has the Potential for Diagnostic Applications. Diagnostics.

[62] Campos G.S., Bandeira A.C., Sardi S.I. (2015). Zika Virus Outbreak, Bahia, Brazil. Emerg. Infect. Dis..

[63] Kuno G. (2003). Serodiagnosis of flaviviral infections and vaccinations in humans. Adv. Virus Res..

[64] Gromowski G.D., Barrett A.D. (2007). Characterization of an antigenic site that contains a dominant, type-specific neutralization determinant on the envelope protein domain III (ED3) of dengue 2 virus. Virology.

[65] Sankar S.G., Balaji T., Venkatasubramani K., Thenmozhi V., Dhananjeyan K., Paramasivan R., Tyagi B., Vennison S.J. (2014). Dengue NS1 and prM antibodies increase the sensitivity of acute dengue diagnosis test and differentiate from Japanese encephalitis infection. J. Immunol. Methods.

[66] Hall R.A., Kay B.H., Burgess G.W., Clancy P., Fanning I.D. (1990). Epitope analysis of the envelope and non-structural glycoproteins of Murray Valley encephalitis virus. J. Gen. Virol..

[67] Falconar A.K., Young P.R. (1990). Immunoaffinity purification of native dimer forms of the flavivirus non-structural glycoprotein, NS1. J. Virol. Methods.

[68] Albinsson B., Rönnberg B., Vene S., Lundkvist Å. (2019). Antibody responses to tick-borne encephalitis virus non-structural protein 1 and whole virus antigen—A new tool in the assessment of suspected vaccine failure patients. Infect. Ecol. Epidemiology.

[69] Stiasny K., Leitner A., Holzmann H., Heinz F.X. (2021). Dynamics and Extent of Non-Structural Protein 1-Antibody Responses in Tick-Borne Encephalitis Vaccination Breakthroughs and Unvaccinated Patients. Viruses.

[70] Albinsson B., Vene S., Rombo L., Blomberg J., Lundkvist Å., Rönnberg B. (2018). Distinction between serological responses following tick-borne encephalitis virus (TBEV) infection vs vaccination, Sweden 2017. Euro Surveill. Bull. Eur. Sur Mal. Transm. Eur. Commun. Dis. Bull..

[71] Girl P., Bestehorn-Willmann M., Zange S., Borde J.P., Dobler G., von Buttlar H. (2020). Tick-Borne Encephalitis Virus Nonstructural Protein 1 IgG Enzyme-Linked Immunosorbent Assay for Differentiating Infection versus Vaccination Antibody Responses. J. Clin. Microbiol..

[72] Dobler G., Euringer K., Kaier K., Borde J.P. (2023). Serological Protection Rates against TBEV Infection in Blood Donors from a Highly Endemic Region in Southern Germany. Vaccines.

[73] Salat J., Mikulasek K., Larralde O., Formanova P.P., Chrdle A., Haviernik J., Elsterova J., Teislerova D., Palus M., Eyer L. (2020). Tick-Borne Encephalitis Virus Vaccines Contain Non-Structural Protein 1 Antigen and May Elicit NS1-Specific Antibody Responses in Vaccinated Individuals. Vaccines.

